# Current Concepts in the Management of Rheumatoid Hand

**DOI:** 10.1155/2015/648073

**Published:** 2015-07-08

**Authors:** Umile Giuseppe Longo, Stefano Petrillo, Vincenzo Denaro

**Affiliations:** ^1^Department of Orthopaedic and Trauma Surgery, Campus Bio-Medico University, Via Alvaro del Portillo 200, Trigoria, 00128 Rome, Italy; ^2^Integrate Centre of Research (CIR), Campus Bio-Medico University, Via Alvaro del Portillo 21, 00128 Rome, Italy

## Abstract

Rheumatoid arthritis (RA) is a chronic inflammatory disease caused by a T cell-driven autoimmune process, which majorly involves the diarthrodial joints. It affects 1% of the US population, and approximately 70% of patients with RA develop pathologies of the hand, especially of the metacarpophalangeal joints (MCP). Furthermore, also the extensor and flexor tendons of the fingers are frequently involved. The first line of treatment should be conservative. Three general classes of drugs are currently available for RA: nonsteroidal anti-inflammatory agents (NSAIDs), corticosteroids, and disease modifying antirheumatic drugs (DMARDs). Encouraging results have been obtained using DMARDs. However, when severe deformities occur or when patients are unresponsive to medical management and injections therapy, surgical intervention should be performed to relieve pain and restore function. Several surgical options have been described for the management of MCP joint deformities, including soft tissue procedures, arthrodesis, and prosthetic replacement. Tendons ruptures are generally managed with tendon transfer surgery, while different surgical procedures are available to treat fingers deformities. The aim of the present review is to report the current knowledge in the management of MCP joint deformities, as well as tendons damage and fingers deformities, in patients with RA.

## 1. Introduction

Rheumatoid arthritis (RA) is a chronic inflammatory disease caused by a T cell-driven autoimmune process, which majorly affects the diarthrodial joints. Women are involved four times more than men, between 35 and 45 years of age [1].

Approximately, 70% of patients with RA develop pathologies of the hand, especially of the metacarpophalangeal joints (MCP). Besides, tenosynovitis and tendon ruptures are also frequent [2, 3]. Joint damage and tendon ruptures are common in patients with RA, leading to severe deformities that hinder the ability to grip, grasp, and pinch. Patients often report a reduction of their quality of life due to inability to perform several activities of daily living.

The first line of treatment should be conservative. Three general classes of drugs are used in the treatment of RA: nonsteroidal anti-inflammatory agents (NSAIDs), corticosteroids, and disease modifying antirheumatic drugs (DMARDs) [4]. Nonsteroidal anti-inflammatory drugs (NSAIDs) produced good results in terms of pain relief and reduction of joint inflammation [5], while corticosteroids regulate immune system activity when NSAIDs are no longer able to control the symptoms [6]. Nevertheless, multiple adverse side-effects ranging from mild irritability to severe and life-threatening cardiovascular events and adrenal insufficiency are associated with the prolonged use of corticosteroids [6]. Moreover, both NSAIDs and corticosteroids are not able to change the disease course or help to improve radiographic outcomes. Only DMARDs showed the capacity to reduce the activity of RA improving also the radiographic outcomes [4, 7]. These can be nonbiologic and biologic. The most common nonbiologic DMARD is methotrexate, which represented the gold standard for treating RA patients until the production of biological agents. On the other hand, biologic agents can be divided into two subgroups: tumor necrosis factor (TNF) inhibitors and interleukin-1 receptor antagonists [8]. Both classes of drugs reduce the cytokines' activity modulating the inflammatory process that underlies RA pathogenesis, and encouraging results in terms of radiographic progression and function have been reported in the literature [9]. However, when joint damage occurs, determining severe deformities, or when patients are unresponsive to medical management and injections therapy, surgical intervention should be considered.

The aim of this paper is to report the current concepts in the surgical management of rheumatoid hand.

## 2. MCP Joints

The most frequent deformity of the hand occurring in patients with RA affects the MCP joint and it is characterized by a volar subluxation of the proximal phalanges and ulnar drift of the fingers [10]. This ulnar deviation of the MCP joint is usually caused by the chronic synovitis, which disrupts the ligamentous support of the joint [10]. Consequently, the radial stress on the fingers with pinch drives the fingers in the ulnar direction. Patients presenting with this deformity often report inability to extend the fingers. Moreover, the deformity limits the ability to cup the fingers around larger objects, and fine pinch is obstructed because the index and middle fingers can no longer oppose the thumb in a tip-to-tip pinch.

The deformities of the MCP joint in patients with RA represent one of the most challenging situations to treat in hand surgery. MCP joint activity is crucial in the arc of motion of the finger, which is initiated at the MCP joint. For this reason, fusion of the finger at the MCP joint is rarely performed [11]. Despite the aesthetic advantage reached after the fusion of the MCP, the loss of motion can be too much disabling, impairing patient's activities of daily living.

Synovectomy of the MCP associated with a crossed intrinsic transfer, in which the ulnar lateral bands are transferred to either the proximal phalanges or the extensor tendons, has been advocated as a good solution in the early stages of RA [11]. This procedure restores the posture of the finger but its feasibility is limited because it can be performed only if the subluxed fingers can be easily reduced to the anatomical position. Furthermore, if there is an ulnar deviation deformity of the MCP joint but there is no damage of the articular surface and there is no volar subluxation, the released radial intrinsic collateral ligament can be performed for a crossed intrinsic transfer to correct the ulnar drift deformity [12]. Soft tissues procedures are less invasive and associated with shortened recovery. However, in case of MCP destruction or when a chronic MCP subluxation is present, arthroplasty procedure is necessary to relieve pain and restore function.

MCP joint arthroplasty is a valuable surgical option for the management of severe deformities of the MCP joint [13]. It is indicated if there is damage of the articular surface of the MCP joint or in case of severe volar subluxation. Shortening the finger, the MCP joint arthroplasty reduces the tension on the tendons and ligaments contributing to the ulnar deformity. Several studies showed good short-term functional and aesthetic outcomes of the silicone metacarpophalangeal joint arthroplasty (SMPA) procedures [14–16]. Despite these encouraging results, high rate of breakage of SMPA has been reported at a long-term follow-up [17]. Probably the silicone material of SMPA is not such resistant at a long term.

Tupper [18] described the volar plate arthroplasty procedure before the availability of the SMPA. Currently, volar plate arthroplasty procedure is used when the medullary cavity is too small to accommodate an implant. The proximal volar plate is detached and sutured to the dorsum of the metacarpal in order to provide a buffer between the bone ends. Nevertheless, this procedure is associated with a high rate of deviation.

Recently, unconstrained two-piece pyrocarbon implants for the rheumatoid MCP joint have been proposed. These implants have been developed for the osteoarthritic MCP joints, demonstrating good results in terms of pain relief and function. However, their use in RA may be a risk because of ligament alteration [19]. The ligamentous alteration of patients with RA leads to joint instability, which may produce an early failure of the two-piece pyrocarbon implants.

## 3. Tenosynovitis and Tendon Ruptures

Synovitis is the hallmark of patients with RA. Tenosynovitis of the extensor tendons of the hand is more frequent than the flexor tendons [20]. Diffuse tenosynovitis is present in a relevant percentage of patients (30%) [1].

It has been shown that chronic synovitis changes the structural properties of the tendons, producing degenerative changes leading to loss of function and rupture without additional mechanical irritation. Moreover, the typical bony prominences found in patients with RA may be responsible of abrasion of the tendons and consequent rupture [21].

From a clinical viewpoint, patients with tenosynovitis of the hand usually present pain and swelling of the palmar aspect of the digit. Palpable crepitus on active and passive flexion of the digit along the flexor tendon and decreased active range of motion are also frequent. Furthermore, patients cannot fully flex the interphalangeal (IP) joints actively, while full passive flexion is possible.

The management of tenosynovitis consists first in tenosynovectomy, which is safe and effective in restoring function in patients with RA. Besides, it can be useful in prevention of tendon ruptures and median nerve compressions [22]. Nevertheless, despite its safety and efficacy, tenosynovectomy of the flexor tendons of the hand may produce severe complications such as injuries of the digital neurovascular bundles or of the median nerve.

The ruptures of the extensor tendons of the fingers are frequently seen in patients with RA. Generally, their management consists in tendon transfer procedures, and several different surgical options have been described [23]. The ruptures of the extensor tendon of the little finger can be managed transferring the distal end of the intact tendon end-to-side to the intact ring finger. However, in case of rupture of the extensor tendon of both ring and little fingers, the extensor indicis proprius tendon can be transferred to power the ring and little fingers. When the middle, ring, and little fingers are ruptured, the extensor indicis proprius tendon is transferred to power the ring and little fingers and the middle finger extensor tendon can be transferred to the index communis extensor tendon. Flexor tendons transfers have been also described for the management of extensor tendons ruptures. The FDS tendon of the middle or ring finger can be transferred to power the ring and little fingers, while the extensor indicis proprius tendon is used to power the middle finger. When all four extensor tendons are ruptured, the tendon transfer option requires the use of both of the FDS tendons from the middle and ring fingers to power the index/middle and ring/little fingers, respectively [19].

Flexor tendons ruptures are also common in patients with RA [24, 25]. The ruptures of the FDS can be managed excising the FDS and carrying out a tenosynovectomy of the FDP, while ruptures of the FDP are more difficult to treat [26]. In case of rupture of the FDP distally to the FDS insertion, the advance and repair mechanism is possible as the proximal end is usually caught at the FDS chiasm. However, when the rupture occurs proximally to the FDS insertion, the best treatment option remains the tenodesis or the arthrodesis of the distal IP joint.

Combined ruptures of the FDS and FDP can be managed with two different surgical procedures [27]. The first option is to perform a flexor tenosynovectomy followed by insertion of a silicon rod. Then at a later date, tendon graft procedure should be performed. The second option consists in fusion of the IP joints and suturing one of the long flexors to the base of the proximal phalanx. This second option should increase the strength of flexion at the MCP joint [27].

## 4. Fingers Deformities

The swan neck finger (SN) and the Boutonniere (BN) finger are the most common deformities detected in patients with RA [28]. The SN deformity (SND) is characterized by a flexion of both MCP and distal interphalangeal (DIP) joints associated with the hyperextension of the PIP joint [29]. On the other hand, BN deformity (BND) is characterized by extension of both MCP and DIP joints associated with a flexion deformity of the PIP joint [30].

The SND represents the result of pathology involving MCP, PIP, and DIP joints and the wrist [31, 32]. In order to manage properly the SND, it is important to identify which joint is primarily responsible for the development of the deformity.

Synovitis of the MCP joint causes alteration of the volar plate, determining MCP joint subluxation. At the end it produces a shortening of the intrinsic muscles leading to PIP joint hyperextension. Moreover, synovitis of the MCP joint produces an alteration of the insertion of the long extensors on the dorsal base of the proximal phalanx, producing hyperextension of the PIP joint. The deformity of the PIP joint is the result of the transfer of the extensor force to the base of the middle phalanx. Synovitis of the PIP joint can extend to FDS tendons and joint collateral ligaments. This synovitis produces degeneration of the FDS insertion and of the volar plate and collateral ligaments. It ultimately determines abnormal hyperextension of the PIP joint because of the action of the extensor forces [33, 34]. Synovitis of the DIP joint produces the rupture of the terminal extensor tendon insertion. Mallet finger is the result of the synovitis. Consequently, the volar supporting structures of the PIP joint are damaged because the extensor forces are modified, leading to PIP joint hyperextension. Synovitis at the wrist has been also described as a possible cause of SND development. It may produce MCP joint flexion and PIP joint extension because the carpal collapse secondary to wrist synovitis leads to a lengthening of the long flexor and extensor tendons. In this manner the interosseous muscle produces the MCP joint flexion and the PIP joint extension.

Usually, deformities of the DIP joint are managed with DIP fusion. On the other hand, several different surgical procedures have been described for the treatment of the hyperextension deformity of the PIP joint. When a flexible hyperextension deformity of the PIP joint is present, the most accredited surgical option considered is tenodesis, which can be performed using the FDS [35] or the conjoint lateral band [36]. Despite the fact that the conjoint lateral band tenodesis allows correction of both hyperextension deformity of the PIP joint and flexion deformity of the DIP joint, it can be challenging to perform due to variability of the anatomy of these structures in patients with RA. Soft tissue release of the hyperextended PIP joint, consisting in the mobilization of the dorsally displaced conjoint lateral bands, has been also proposed in few selected and compliant patients. After the treatment of the PIP joint, it is crucial to treat both MCP and DIP joint deformities, which may contribute to hyperextension of the PIP joint [35]. In patients with severe damage (i.e., articular cartilage surface damaged; unstable dislocations; and irreducible hyperextension deformity) of the PIP joint, arthroplasty or fusion is indicated. Both surgical options demonstrated good results in terms of pain relief, function, and aesthetic satisfaction. Nevertheless, because of alteration of ligamentous supports of the PIP joint which is typical in patients with RA, arthroplasty does not present enough stability and it is associated to high rate of failure. For this reason, fusion of the PIP joint in flexion remains the much safer procedure [37].

The BND is caused by intra-articular proliferation of the synovia of the PIP joint, which distends the capsuloligamentous apparatus, leading to extension of both MCP and DIP joints associated with a flexion deformity of the PIP joint [34]. Furthermore, the synovial pannus produces erosions of the collateral ligaments with consequent detachment. The synovium also stretches the conjoint lateral bands laterally, breaking through between the central band and the conjoint lateral bands [30].

The surgical treatment of BND represents a challenge for hand surgeons. Despite the fact that BND is not disabling as SND and rarely compromises PIP joint function, several surgical procedures have been described for the management of this deformity. In the early stage of BND, when a full passive range of motion of PIP joint is present, the PIP joint can be extended with a splint. However, it is important to extend only the PIP joint without extending also the DIP joint. In case of persistent synovitis of the PIP joint unresponsive to oral medications and injective therapy, synovectomy should be performed. In patients with limited passive range of motion of the PIP joint, complete passive correction of the flexion deformity using a splint is often impossible due to irreversible soft tissue changes. In these cases, surgery aims to restore extensor force to the PIP joint. This can be performed by repairing the central band, maintaining the conjoint lateral bands dorsally. However, this procedure limits PIP joint flexion [38]. The reconstruction of the extensor tendon has been also described. Some authors proposed to perform it under digital block anesthesia. In this manner the patients can participate during surgery, extending and flexing the finger.

Patients with no passive motion of the PIP joint have irreversible soft tissue contractures and erosions of articular surface. In some cases, ankylosis is present. Surgical options in these cases are joint arthrodesis or arthroplasty. However, the use of arthroplasty for the management of BND is limited because it is necessary to significantly resect the proximal phalanx, leading to collateral ligaments instability. Moreover, the reconstruction of the extensor tendon is necessary, determining a delayed mobilization of the PIP joint [38]. For these reasons, fusion should be preferred to arthroplasty to manage patients with RA affected by BND.

## 5. Conclusions

The management of rheumatoid hand requires an integrated approach involving both rheumatologist and orthopaedics surgeons. The first line of treatment should be conservative, including medical (NSAIDs; corticosteroids; DMARDs) and injective therapy. Excellent clinical and radiographic results in patients with mild deformities or tenosynovitis have been obtained since the introduction of DMARDs. However, when patients develop severe deformities with no passive range of motion of the joint affected, surgery is necessary. Furthermore, tenosynovitis unresponsive to medical treatment, as well as tendon ruptures, needs surgical intervention.

Several different surgical options are available for MCP joint deformities such as SND and BND. A correct clinical examination focused on the evaluation of passive range of motion of the joint involved is crucial to plan surgery. Soft tissues procedures represent the gold standard for the management of SND and BND if a passive range of motion of the PIP joint is present. Nevertheless, in case of ankyloses of the PIP joint or when a full passive range of motion of the PIP joint is absent, the available treatment options are arthroplasty or fusion.
